# Seasonal Weather Changes Affect the Yield and Quality of Recombinant Proteins Produced in Transgenic Tobacco Plants in a Greenhouse Setting

**DOI:** 10.3389/fpls.2019.01245

**Published:** 2019-10-08

**Authors:** Matthias Knödler, Clemens Rühl, Jessica Emonts, Johannes Felix Buyel

**Affiliations:** ^1^Bioprocess Engineering, Fraunhofer Institute for Molecular Biology and Applied Ecology IME, Aachen, Germany; ^2^Institute for Molecular Biotechnology, RWTH Aachen University, Aachen, Germany

**Keywords:** batch reproducibility, environmental correlation, fluorescent protein carrier, greenhouse cultivation, plant molecular farming, protease activity

## Abstract

Transgenic plants have the potential to produce recombinant proteins on an agricultural scale, with yields of several tons per year. The cost-effectiveness of transgenic plants increases if simple cultivation facilities such as greenhouses can be used for production. In such a setting, we expressed a novel affinity ligand based on the fluorescent protein DsRed, which we used as a carrier for the linear epitope ELDKWA from the HIV-neutralizing antibody 2F5. The **D**sRed-2**F**5-**e**pitope (DFE) fusion protein was produced in 12 consecutive batches of transgenic tobacco (*Nicotiana tabacum*) plants over the course of 2 years and was purified using a combination of blanching and immobilized metal-ion affinity chromatography (IMAC). The average purity after IMAC was 57 ± 26% (n = 24) in terms of total soluble protein, but the average yield of pure DFE (12 mg kg^−1^) showed substantial variation (± 97 mg kg^−1^, n = 24) which correlated with seasonal changes. Specifically, we found that temperature peaks (>28°C) and intense illuminance (>45 klx h^−1^) were associated with lower DFE yields after purification, reflecting the loss of the epitope-containing C-terminus in up to 90% of the product. Whereas the weather factors were of limited use to predict product yields of individual harvests conducted for each batch (spaced by 1 week), the average batch yields were well approximated by simple linear regression models using two independent variables for prediction (illuminance and plant age). Interestingly, accumulation levels determined by fluorescence analysis were not affected by weather conditions but positively correlated with plant age, suggesting that the product was still expressed at high levels, but the extreme conditions affected its stability, albeit still preserving the fluorophore function. The efficient production of intact recombinant proteins in plants may therefore require adequate climate control and shading in greenhouses or even cultivation in fully controlled indoor farms.

## Highlights

- DsRed is a strongly expressed carrier for linear epitope ligands.
- Fusion protein accumulation in transgenic plants is affected by seasonal weather changes.
- Temperature and illuminance peaks during cultivation compromise product integrity.
- Temperature and illuminance peaks trigger an increase in endogenous protease activity.
- The timing of temperature/illuminance stress affects the severity of product degradation.

## Introduction

Plants have been developed as expression systems for the production of recombinant proteins including biopharmaceuticals (Hiatt et al., 1989), some of which are entering clinical trials (Ma et al., 2015), and a few are already on the market (Mor, 2015). Plant-based expression systems offer pharmaceutical companies several advantages compared to traditional mammalian cell culture platforms, including lower upstream production costs, better intrinsic safety, and greater scalability (Buyel et al., 2015; Sack et al., 2015; Spiegel et al., 2018). The scalability of plants is especially appealing if agricultural infrastructure can be used because this would provide sufficient capacity to produce several tons of purified protein per year (Stoger et al., 2002; Buyel et al., 2017). However, the risk of contamination is elevated by the abundance of pathogens, animals, and agrichemicals in the open field, so fully contained facilities have been designed that allow the controlled cultivation of plants on a medium to large scale (Wirz et al., 2012; Holtz et al., 2015). Such facilities require substantial upfront investment and operate under complex and thus error-prone process control systems, which may offset some of the cost savings achieved by switching from mammalian cells to plants. Greenhouses offer an attractive compromise because they achieve sufficient containment with only moderate infrastructure costs, as shown by the use of greenhouse facilities to cultivate plants expressing a monoclonal antibody that was purified and used in phase I clinical trials (Ma et al., 2015; Sack et al., 2015).

**Figure 1:**
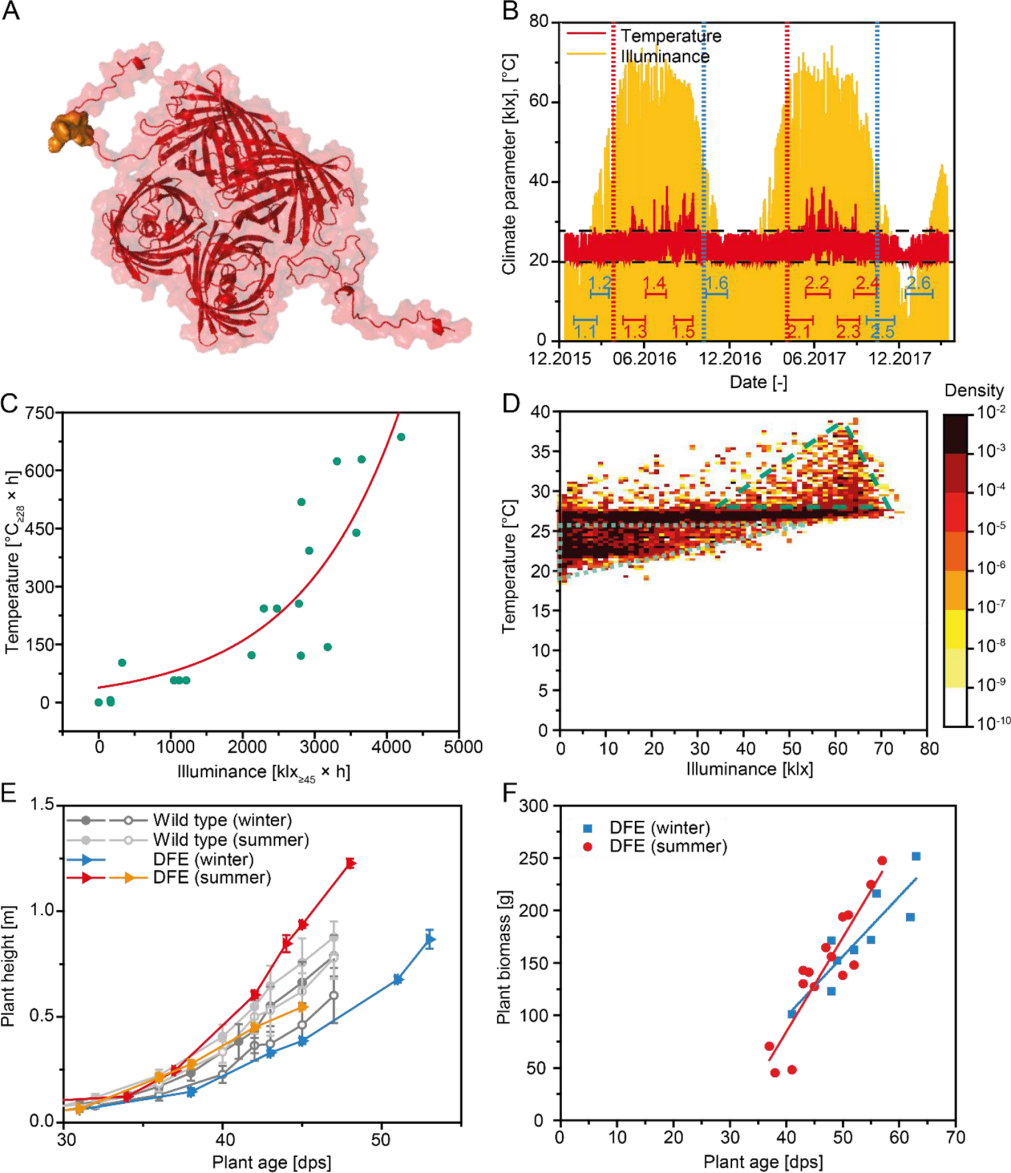
Climate data and plant growth during the production of DFE. **(A)** Three-dimensional structure of a DFE tetramer. The DsRed part is shown in red, and the 2F5 epitope of the fusion part consisting of the 2F5 epitope, His_6_ tag, and KDEL tag is highlighted in gold. **(B)** Temperature and illuminance data for the duration of this study. Batch durations are marked with double-T lines and numbers (blue = winter, red = summer). Vertical lines indicate the transition between seasons (blue = winter, red = summer). Dashed horizontal lines indicate temperature control limits. **(C)** Correlation between integrated temperature >28°C and integrated illuminance >45 klx approximated by a two-parameter exponential model. **(D)** Heat map of ∼20,000 temperature–illuminance data points recorded with a frequency of one measurement per hour during 12 batches shown in **(B)** The dotted triangle marks data well within the temperature control range whereas the dashed triangle indicates a region with measurements outside the control limits. **(E)** Plant height according to plant age observed during cultivation in winter or summer for transgenic plants expressing DFE as well as wild-type controls. Error bars indicate the standard deviation (n ≥ 10); individual lines correspond different batches. **(F)** Average plant biomass at the time of harvest according to the age of transgenic plants expressing DFE. Six data points were obtained from a first set of six batches, whereas 18 data points were collected from a second set of six batches for which plants were harvested at three time points each. Lines indicate linear regression model on the data for winter (n = 9, r = 0.89) and summer (n = 15, r = 0.91) harvests.

One drawback of greenhouse cultivation is the incomplete control of environmental conditions such as temperature and light, but the effects of these parameters on recombinant protein expression have not been considered in detail. Here, we describe the results of a long-term study in which transgenic tobacco (*Nicotiana tabacum* cv. Petit Havana SR1) plants expressing a recombinant fusion protein were cultivated in 12 consecutive batches in a greenhouse setting over the course of 2 years. The fusion protein comprised the fluorescent marker protein DsRed (Baird et al., 2000) with a C-terminal extension featuring a linear epitope (ELDKWA in the one-letter amino acid code) from the HIV-neutralizing antibody 2F5 (Muster et al., 1993; Parker et al., 2001), a His_6_ affinity tag, and a KDEL tag for retrieval of the protein to the endoplasmic reticulum. The product was named DFE, for **D**sRed-2**F**5-**E**pitope (**Figure 1A**) (Rühl et al., 2018). We monitored the accumulation of DFE in 12 batches at several growth stages and also recorded the absolute product yield, the recovery after purification, and seasonally dependent protease activity reflecting the changing climate in the greenhouse. We discuss the impact of these environmental parameters on the production of recombinant proteins in plants cultivated in a greenhouse setting.

## Materials and Methods

### Plant Material and Cultivation

Transgenic tobacco plants (*N. tabacum* cv. Petit Havana SR1) expressing DFE were generated as previously described (Buyel et al., 2014) and bred to the fifth generation (T_5_) by self-pollination to produce seeds for further experiments. The T_5_ plants were cultivated in a greenhouse at the Fraunhofer Institute for Molecular Biology and Applied Ecology IME, Aachen, Germany (50°47′07.1″N 6°03′00.5″E) from 21 December 2015 to 6 February 2018. The plants were grown in soil and were irrigated with 0.1% (m/v) Ferty 2 MEGA (Gärtnereibedarf Kammlott GmbH, Erfurt, Germany). The temperature in the greenhouse was set to a maximum of 27°C and a minimum of 22°C (day) and 20°C (night) with artificial auxiliary lighting (400 W) provided by MASTER HPI-T Plus quartz metal halide and MASTER Agro high-pressure sodium lamps (Koninklijke Philips, Amsterdam, Netherlands) distributed so that 0.75 lamps of each type were provided per m^2^ cultivation area (9.5 klx, corresponding to ∼180 μmol s^−1^ m^−2^ according to the manufacturer’s data; λ = 400–700 nm). The lights were activated if illuminance fell below 50.0 klx during the 16-h photoperiod. The relative humidity was set to 50% with a control range of 30–70% and measured with digital hygrometers inside and outside of the greenhouse. Additionally, outside wind velocity (m s^−1^) and daily precipitation (mm) were measured using a digital anemometer and a digital hyetometer, respectively, both positioned outside at the greenhouse gable. The plants were cultivated for 36–63 days depending on their size before harvest. A single harvest of 10 plants was conducted for the first six batches, and their leaves were subjected to extraction. For batches 7–12, additional harvests of up to 10 plants were conducted 1 week before and 1 week after the main harvest, increasing the total number of plants up to 30 per batch.

### Protein Extraction and Clarification

Total soluble protein (TSP) was extracted from 3 to 10 kg of leaves after blanching (Rühl et al., 2018) by maceration in a blade-based homogenizer containing 3 L of extraction buffer (50 mM sodium phosphate, 500 mM sodium chloride, 10 mM sodium bisulfite, pH 8.0) per kilogram of wet biomass, as previously described (Buyel and Fischer, 2014a). The extract was clarified by passage through a series of bag, depth, and sterile filters (Buyel and Fischer, 2014).

### Purification of DFE by Chromatography

DFE was purified on an ÄKTApure system (GE Healthcare, Uppsala, Sweden) fitted with an XK-26 column containing 53 ml of chelating Sepharose fast-flow-immobilized metal-ion affinity chromatography (IMAC) resin loaded with nickel ions and pre-conditioned with extraction buffer lacking sodium bisulfite. After loading the clarified extract, unbound proteins were washed through with 10 column volumes of the same buffer, and bound proteins were then eluted in the same buffer supplemented with 300 mM imidazole at a flow rate of 50 cm h^−1^. The protein and nucleic acid concentrations were monitored at 280 and 260 nm, respectively.

### Protein and Product Quantitation

The TSP concentration was determined using a microtiter plate version of the Bradford method (Buyel and Fischer, 2014), and the protein composition was analyzed by lithium dodecylsulfate (LDS) polyacrylamide gel electrophoresis (PAGE) followed by gel staining with Coomassie Brilliant Blue (Menzel et al., 2016). DFE was quantified by fluorescence spectroscopy against DsRed standards (Buyel and Fischer, 2012). Briefly, DsRed fluorescence in the clarified extracts was measured using a Synergy HT microplate reader (BioTek Instruments, Winooski, Vermont, USA) fitted with 530/25 (excitation) and 590/35 (emission) nm filter sets. A standard curve was prepared with DsRed dilutions in the range 0–225 mg L^−1^, and the protein accumulation level per gram wet biomass was calculated as described elsewhere (Gengenbach et al., 2018). The presence of the C-terminal 2F5-epitope (ELDKWA) and His_6_ tag was confirmed by immunoblotting using an in-house preparation of the human monoclonal antibody 2F5 (Rühl et al., 2018) and a monoclonal rabbit anti-His_6_ antibody (BioVision, Milpitas, California, USA), respectively. These primary antibodies were detected using polyclonal goat-anti-human and goat-anti-rabbit immunoglobulin secondary antibodies, respectively, each conjugated to alkaline phosphatase (Jackson ImmunoResearch, Cambridge, UK).

### Measurement of Protease Activity

The protease activity in plant extracts after blanching heat treatment and in untreated controls was determined using a colorimetric protease assay (Thermo Fisher Scientific, Waltham, Massachusetts, USA) according to the manufacturer’s instructions. Samples were diluted in the range 1:5–1:40 to adjust the TSP concentrations to the same order of magnitude and were then measured in triplicate in 96-well plates. Six trypsin standards with concentrations of 0.005–500 mg L^−1^ were used to build duplicate standard curves. For each sample and standard, 100 µl of succinylated casein solution was pipetted into one well and 100 µl of working buffer into another as a blank, before transferring 50 µl of the sample to both. The plates were incubated for 20 min at 22°C before adding 50 µl of working solution to each well and were then incubated for an additional 20 min at 22°C. The absorbance in each well was measured twice at 450 nm using an EnSpire multimode plate reader, and the data were exported in EnSpire Manager v4.13 (Perkin Elmer, Waltham, Massachusetts, USA).

### Data Analysis

Key figures were calculated for each weather factor (K_wf_) for the entire growth period as well as for individual growth phases [germination up to 0–14 days post-seeding (dps), sprouting 15–24 dps, growth 25–38 dps, and maturation 38 dps to harvest] as shown in Equation 1, where *m* is the number of data points (one value for every hour during cultivation), *wf_act_* is the actual value of the weather factor at time point *j*, and *wf_set_* is the set point or critical value of the weather factor.

(1) $$K_{\mathrm{wf}}=\sum_{j=1}^{m}\left({\mathrm{wf}}_{\mathrm{act}}-{\mathrm{wf}}_{\mathrm{set}}1({\mathrm{wf}}_{\mathrm{act}}>{\mathrm{wf}}_{\mathrm{set}}\right)$$

Averages of the weather factors and their key figures were calculated for the total cultivation period and for the maturation phase.

The sample Pearson correlation coefficient *r_XY_* (Equation 2) was used to describe the correlation between two variables, where {(*x*_1_, *y*_1_), (*x*_2_, *y*_2_), …,(*x*_n_, *y*_n_)} are *n* given data pairs, and $\bar{x}$ and $\bar{y}$ are the sample averages.

(2) $$\begin{array}{c} r_{\mathrm{XY}}=\;\frac{\sum_{i=1}^{n}\left(x_{i}-\bar{x}\right)\left(y_{i}-\bar{y}\right)}{\sqrt{\sum_{i=1}^{n}{\left(x_{i}-\bar{x}\right)}^{2}}\sqrt{\sum_{i=1}^{n}{\left(y_{i}-\bar{y}\right)}^{2}}},\\ \bar{x}=\frac{1}{n}\sum_{i=1}^{n}x_{i},\bar{y}=\frac{1}{n}\sum_{i=1}^{n}y_{i} \end{array}$$

The significance of the null-hypothesis ρ*_XY_* = 0 was tested based on variable *t*, which is characterized by Student’s *t*-distribution with *n* − 2 degrees of freedom (Equation 3).

(3) $$t=\;\frac{r_{\mathrm{XY}}\cdot \sqrt{n-2}}{\sqrt{1-r_{\mathrm{XY}}^{2}}}$$

Thus, Student’s *t*-distribution provides the probability (*p*-value) to observe a value for *t* that is at least as large as a critical *t* value for a specific significance level α (here, α = 0.05). This is equivalent to the probability of finding a correlation coefficient *r_XY_* at least as large as that used for the underlying calculation of *t* given that the true correlation is zero. We considered *p*-values <5% as interesting and *p*-values <1% as significant.

Partial correlations between two variables *X* and *Y* without the effect of a third variable *U* were found by first establishing a linear regression between *X* or *Y* and *U* and then calculating the correlation between the residues (Equation 4).

(4) $$r_{\mathrm{XY}\cdot U}=\;\frac{r_{\mathrm{XY}}-r_{\mathrm{XU}}\cdot r_{\mathrm{YU}}}{\sqrt{1-r_{\mathrm{XU}}^{2}}\sqrt{1-r_{\mathrm{YU}}^{2}}}$$

We calculated the significance of the partial correlation using the test statistic t* (Equation 5), which is also characterized by Student’s *t*-distribution, but this time with n − 3 degrees of freedom.

(5) $$t^{*}=\frac{r_{\mathrm{XY}\cdot U}\cdot \sqrt{n-3}}{\sqrt{1-r_{\mathrm{XY}\cdot U}^{2}}}$$

A multiple linear model was used to describe dependent variable *Y* by *q* independent variables *X*_1_, *X*_2_, …, *X*_q_ by means of a linear function with disturbance ϵ (Equation 6).

(6) $$Y=\;\beta_{0}+\beta_{1}X_{1}+\ldots +\;\beta_{q}X_{q}+\epsilon$$

The model coefficients were estimated by minimizing the sum of squared differences between the predicted and observed values (ordinary least squares, Equation 7) with {(*x*_11_, …, *x*_q1_, *y*_1_), (*x*_12_, …, *x*_q2_, *y*_2_), …,(*x*_1n_, …, *x*_qn_, *y*_n_)} *n* data-tuples.

(7) $$\sum_{i=1}^{n}{\left(y_{i}-\beta_{0}+\beta_{1}x_{1i}+\ldots +\;\beta_{q}x_{\mathrm{qi}}\;\right)}^{2}\to \underset{\beta_{0},\beta_{1},\ldots ,\beta_{q}}{\min}\;$$

The coefficient of determination and the adjusted coefficient of determination for the (multiple) linear regression models were calculated using Equations 8 and 9, respectively.

(8) $$R^{2}=\;1-\;\frac{\sum_{i=1}^{n}{\left(y_{i}-{\hat{y}}_{i}\right)}^{2}}{\sum_{i=1}^{n}\left(y_{i}-\bar{y}\right)},$$

with *y*_1_, …, *y_n_* observed and ${\bar{y}}_{i},\ldots {\bar{y}}_{n}$ predicted values, $\bar{y}=\;\frac{1}{n}\overset{n}{\underset{i=1}{\sum }}y_{i}$

(9) $$\mathrm{adj}.\;R^{2}=R^{2}-\left(1-R^{2}\right)\times \frac{n-1}{\left(n-q-1\right)}$$

The slopes of two linear regression functions were compared using statistic t** (two-sided test, α = 0.05) (Equation 10), where b_1_ and b_2_ are the function slopes, s.e.(b1–b2) is the standard error of the difference between the slopes, S_XX_ is the sum of squared differences between the independent variable and its mean value, SSE is the sum of squared errors, and s^2^ is the pooled estimator of variance.

(10) $$\begin{array}{l} t^{**}=\frac{b_{1}-b_{2}}{s.e.(b_{1}-b_{2})},s.e.\;(b_{1}-b_{2})s^{2}=\sqrt{s^{2}\times \left[\frac{1}{s_{\mathrm{xx},1}}+\frac{1}{s_{\mathrm{xx},2}}\right]\;,}\\ \;s^{2}\;=\frac{{\mathrm{SSE}}_{1}+{\mathrm{SSE}}_{2}}{n_{1}+n_{2}-4},\mathrm{SSE}=\sum_{i=1}^{n}{\left(y_{i}-{\hat{y}}_{i}\right)}^{2},S_{xx}=\sum_{i=1}^{n}{\left(x_{i}-\bar{x}\right)}^{2} \end{array}$$

The relative yield of an individual harvest from one batch was calculated using Equation 11, where ry_i_ is the relative harvest yield with i denoting the harvest time (1—first harvest, 2—second harvest, 3—third [final] harvest), y_i_ is the DFE yield of the harvest in mg kg^−1^ biomass, and $\bar{y}$ is the average yield of one batch.

(11) $${\mathrm{ry}}_{i}=\frac{y_{i}}{\frac{\sum_{i=1}^{n}y_{i}}{n}}=\frac{y_{i}}{\bar{y}}$$

## Results and Discussion

### Greenhouse Climate Control Can Be Insufficient to Maintain Homogeneous Plant Growth During Changing Seasons

Transgenic plants expressing the 28.4-kDa recombinant protein DFE (**Figure 1A**) were cultivated in an initial set of six batches over a period of 12 months covering all seasons of the year. Between April and September (hereafter termed “summer”), intense illuminance and high temperatures externally (average outdoor temperature 15.4°C) resulted in average values of 25.0°C and 8.4 klx inside the greenhouse, compared to 22.7°C and 5.9 klx between October and March (hereafter termed “winter,” average outdoor temperature 6.1°C) (**Figure 1B**). Within the temperature control range of 20 ± 2°C during the night and 25 ± 3°C during the 16-h photoperiod, the temperature correlated with the illuminance (**Figures 1C, D** and **S1A**; adj. R^2^ = 0.89). There were days during the summer when climate control was insufficient to maintain the temperature within the specification limits (**Figures 1B, D**). These out-of-specification temperatures of more than 28°C were associated with an illuminance of >45 klx in 72% of instances (**Figures S1B, C**), indicating that intense sunlight resulted in a greenhouse effect, increasing temperatures in the cultivation area beyond the capabilities of the climate control system.

Plant growth was accelerated under the warm summer conditions reaching a threshold height of 500 mm as early as 40 dps, compared to 47 dps for batches cultivated during the winter (**Figure 1E**). We did not observe any statistically significant differences in growth between the transgenic plants expressing DFE and corresponding *N. tabacum* wild-type controls based on a slope comparison for plant height development [α = 0.05; n = 24 (winter), or n = 15 (summer)]. The biomass yield was positively correlated with plant age (**Figure 1F**), and the slope of this correlation was significantly higher for summer compared to winter batches according to a slope comparison. Our results agreed well with previous reports claiming that the optimal growth temperature for tobacco is in the range 18.5–28.5°C (Parups and Nielsen, 1960; Yamori et al., 2010;Yang et al., 2018).

### Product Recovery by IMAC Purification Is Reduced in Summer Batches

**Figure 2:**
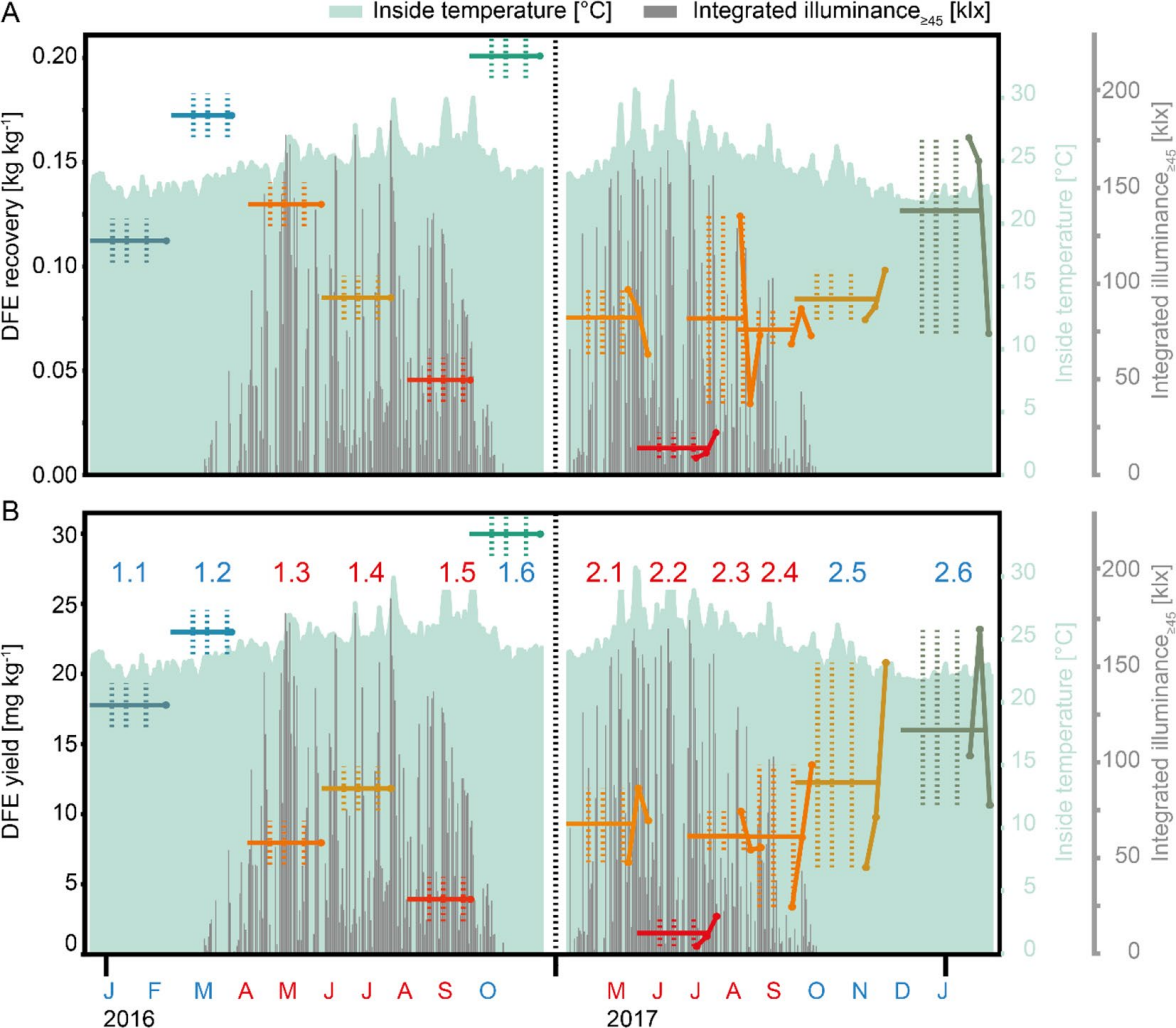
DFE recovery and yield over the changing seasons. **(A)** DFE recovery following IMAC purification. The recovery is defined as the ratio of DFE in the elution fraction (after purification) and the DFE amount in the load (before purification). **(B)** Overall DFE yield after purification per kilogram of fresh plant biomass. Labels in B (numbers) indicate the allocation to summer (red) or winter (blue) batches, and the black vertical dotted lines separate the first (left) and second (right) set of batches. Horizontal lines indicate the average batch recovery **(A)** and yield **(B)** calculated based on all harvests of one batch, whereas colored point-scatter plots correspond to the individual harvest-specific recovery **(A)** and yield **(B)** values in the second batch set. Vertical colored dotted lines mark the transitions between the growth phases in each batch (left line = germination to sprouting, middle line = sprouting to growth, and right line = growth to maturation) analyzed in **Table 2**. Light green areas mark the inside temperature in a greenhouse whereas gray columns correspond to the integrated illuminance.

We did not observe any correlation between biomass and recombinant protein yields, but we found that the recovery and yield of intact full-length DFE after IMAC purification (measured by detecting the presence of the C-terminal epitope and His_6_-tag) varied substantially—for example, falling within the range 3.9–30.0 mg kg^−1^ in the course of 1 year (**Figures 2** and **S1B–D**). In contrast, the yield of a monoclonal antibody expressed in transgenic tobacco plants was previously shown to increase by 25–150% during the summer season (Sack et al., 2015). Interestingly, the specific recovery during the IMAC capture step decreased from ∼35% for winter batches to <10% for summer batches (**Figure S2G**), and we observed substantial fluorescence in the flow-through fraction (**Figure S2E**). One possible explanation was that the DFE conformation was altered in the summer batches such that the His_6_ tag was no longer accessible by the IMAC resin, as reported for other proteins such as erythropoietin (Debeljak et al., 2006). But given that we were unable to detect either the C-terminal 2F5 epitope or His_6_ tag in the IMAC flow-through fraction by western blot analysis even when the samples were denatured (which would expose any linear epitopes hidden by conformational changes), proteolytic degradation potentially triggered by illumination and/or heat stress appeared a more likely explanation (Jutras et al., 2018). Also, compared to the short but intense heating during blanching which causes permanent protease inactivation (Menzel et al., 2018), the heat stress during cultivation was a moderate but long-term (several days) effect which can result in endogenous protease expression. We therefore screened the DFE sequence, especially in the linker region of the fusion protein, for known protease cleavage sites. Using PROSPER prediction software (Song et al., 2012), we found two cleavage sites for cysteine protease cathepsin K close to the C-terminus of the protein at positions 243 and 244 of the 267-amino-acid sequence (N-fragment size = 30.8 kDa) with scores of 1.20 and 1.06, respectively (scores > 0.8 are considered interesting). Proteases of this class have previously been associated with the degradation of recombinant proteins in plants (Niemer et al., 2016). Sites for other proteases were identified in the central region of the protein but cleavage would also abolish the fluorescence of the DsRed parent protein. The detection of fluorescence notwithstanding the loss of the C-terminal epitopes indicated that these sites were not cleaved in our plants.

### Product Yield Is Negatively Correlated With Light Intensity Above 45 klx and Temperature Above 28°C

We then analyzed a second set of six batches based on weather [temperature in °C, illuminance in klx, daily precipitation (rain) in mm, outside wind velocity in m s^−1^, and relative humidity in %], cultivation (plant age and biomass) factors (**Figure S3**), as well as biochemical responses (protease activity, TSP, DFE accumulation, recovery, and yield) from plant samples harvested at three different time points for each batch (**Figures S4** and **S5**). In a first step, we treated the 18 data points (six batches with three harvests each) for each response as independent results, not grouping them by batch.

The protease assay was sensitive to most endoprotease types including serine, acidic, sulfhydryl, and metalloproteases, but we did not observe any correlation between their activity and DFE accumulation levels or yield (**Figure S5**, first column, fourth row). One potential explanation is that the assay sensitivity varies for different protease types, as stated in the manufacturer’s notes, and therefore a change in the activity of one class of proteases might remain unnoticed. Plant age and biomass showed a moderate positive correlation with DFE yield (r = 0.51 and 0.43, respectively) (**Figure S4**, first column, fourth and fifth rows). There was no apparent correlation between internal air humidity and DFE yield, and only a moderate correlation with wind (**Table 1**). A substantial drop in product recovery and yield was observed for increasing temperature and illuminance. Specifically, the DEF yield was on average 50% lower in the summer (6.9 ± 4.2 mg kg^−1^, n = 12) compared to winter batches (14.1 ± 6.6 mg kg^−1^, n = 6), which was a significant difference based on a two-sided two-sample *t*-test (n = 18, α = 0.05, p = 0.045; **Figure 2B**). Interestingly, rain and external humidity were positively correlated with DFE yield to a similar extent as the negative correlation with illuminance. Our interpretation is that these parameters describe the cultivation area shading as a common underlying phenomenon—for example, due to clouds.

**Table 1:** Correlation between climate factors during plant growth and DFE yield.

				24 h		Light period^a^		Dark period^b^	
Data type	Sensor location	Weather factor	Unit	r	p-value	r	p-value	r	p-value
All data	Inside	Temperature	[°C]	−0.709	0.001	−0.705	0.001	−0.685	0.002
		Temperature change	[°C]	−0.644	0.004	−0.665	0.003	−0.586	0.011
		Illuminance	[klx]	−0.686	0.002	−0.686	0.002	n.a.	n.a.
		Relative humidity	[-]	0.140	0.579	0.245	0.327	−0.143	0.572
	Outside	Temperature	[°C]	−0.687	0.002	−0.695	0.001	−0.651	0.003
		Relative humidity	[-]	0.637	0.004	0.641	0.004	0.607	0.008
		Rain	[m]	0.622	0.006	0.595	0.009	0.649	0.004
		Wind	[m s^−1^]	0.522	0.026	0.494	0.037	0.548	0.019
Averages	Inside	Temperature	[°C]	−0.934	0.006	−0.925	0.008	−0.914	0.011
		Temperature change	[°C]	−0.831	0.041	−0.862	0.027	−0.729	0.100
		Illuminance	[klx]	−0.908	0.012	−0.908	0.012	n.a.	n.a.
		Relative humidity	[-]	0.195	0.710	0.329	0.524	−0.168	0.750
	Outside	Temperature	[°C]	−0.894	0.016	−0.907	0.012	−0.840	0.036
		Relative humidity	[-]	0.838	0.037	0.845	0.034	0.790	0.061
		Rain	[m]	0.824	0.044	0.786	0.064	0.867	0.025
		Wind	[m s^−1^]	0.736	0.095	0.702	0.120	0.767	0.075

^a^Daily time between 06:00:00 and 22:00:00; ^b^daily time between 22:00:00 and 06:00:00.

Exploratory data analysis revealed that the DFE yield decreased in line with more frequent, longer, or stronger deviations of illuminance or temperature from the target climate setting. We therefore integrated the temperature above the control threshold of 28°C (Temp_≥28_) and the illuminance above a threshold of 45 klx (Ill_≥45_), which correlated with temperatures above the control threshold (**Figure 1C**) to derive an additional set of key figures. The integration considered either the entire cultivation or different phases, and we correlated the integrated weather factors with the DFE yield (**Table 2**). In all cases, high temperatures and intense light were correlated with a lower DFE yield, and this correlation was significant in some cases—for example, illuminance during germination and sprouting. The correlation of factor averages (normalized for the duration of a phase) was higher than the mere integral. The strongest correlations with DFE yield were found for the average internal temperature over the entire cultivation period (average total, r = −0.709, **Table 1**) and the integrated illuminance >45 klx during sprouting (Ill_≥45,S_, r = −0.716, **Table 2**). The latter implied that events early during cultivation can ultimately have a strong impact on the product yield and thus process performance. Others have identified anthesis as the most heat-sensitive phase in the plant life cycle (Zinn et al., 2010; Zhou et al., 2017; Opole et al., 2018), but this is of limited relevance in molecular farming because plants are typically harvested and processed before flower development (Ma et al., 2015; Sack et al., 2015).

**Table 2:** Correlation between integrated light intensity ≥45 klx or integrated temperature ≥28°C and DFE yield during different growth phases. Average values have been normalized for the duration of the interval between phases.

Cultivation phase		All Data		Averages	
		Integrated illuminance (≥45 klx)	Integrated temperature (≥28°C)	Integrated illuminance (≥45 klx)	Integrated temperature (≥28°C)
Germination	r	−0.598	−0.321	−0.799	−0.428
	p-value	0.009	0.194	0.057	0.397
Sprouting	r	−0.716	−0.647	−0.957	−0.864
	p-value	0.001	0.004	0.003	0.026
Growth	r	−0.480	−0.461	−0.641	−0.616
	p-value	0.044	0.054	0.170	0.193
Maturation	r	−0.296	−0.188	−0.593	−0.430
	p-value	0.233	0.456	0.215	0.394
Entire cultivation	r	−0.609	−0.606	−0.865	−0.881
	p-value	0.007	0.008	0.026	0.020
Maturation (normalized)	r	−0.442	−0.306	−0.668	−0.480
	p-value	0.066	0.217	0.147	0.335
Entire cultivation (normalized)	r	−0.647	−0.629	−0.869	−0.888
	p-value	0.004	0.005	0.025	0.018

**Figure 3:**
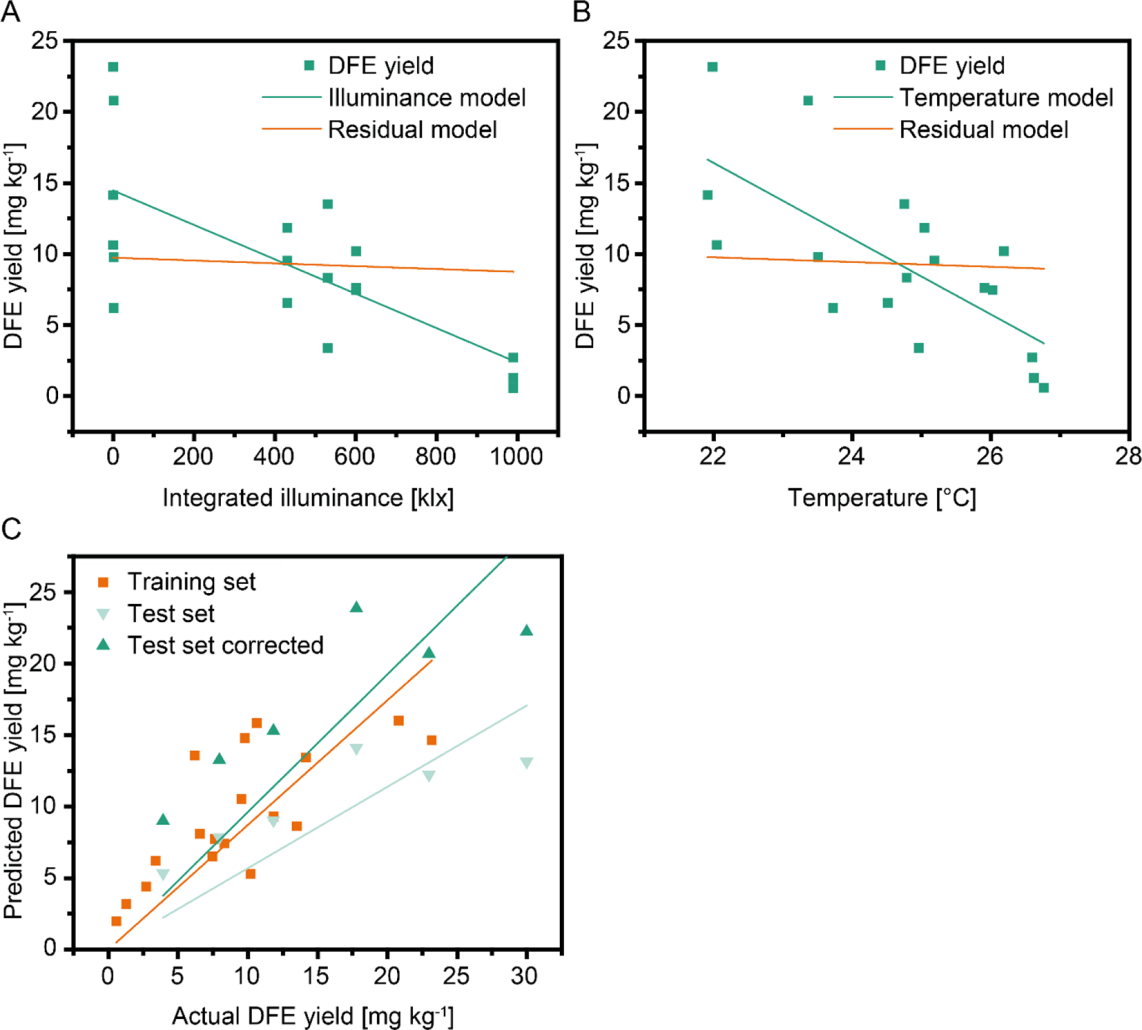
Regression models for DFE yield using different weather factors and cultivation data. The effects of integrated illuminance **(A)** and temperature **(B)** as independent variables were intercorrelated. Green lines indicate the regression model, whereas orange lines correspond to a residual model with the effect of the independent variable removed. A multi linear regression model **(C)** based on integrated illuminance ≥45 klx and plant age showed a high correlation between training (second set of batches, orange line) and test (first set of batches) data. The prediction (light green line) improved substantially (dark green line) if a correlation factor of 1.69 was included in the model to compensate for differences in sample handling between the two sets of batches.

It was not possible to definitively link the DFE yield to either illuminance or temperature due to the high intercorrelation of the two environmental parameters (**Figure 3**). Controlled environments or modified greenhouse settings may help to resolve this collinearity between factors in the future. We extracted additional key figures from the weather factors, such as parameter extrema, number of days outside control ranges, and threshold and mean deviation from the specifications. However, no significant correlations remained after subtracting the effect of Ill_≥45,S_ or Temp_≥28,Av.tot._ by calculating partial correlations between the weather key figures and DFE yield (**Table S1**).

### Increasing Levels of TSP and Protease Activity Are Observed Under Warm Conditions

We also analyzed the key figures described above in the context of DFE accumulation (before purification), DFE recovery, TSP and the protease level, as well correlations among these parameters (**Figure S5**). DFE recovery showed the same correlations as DFE yield (reaching a maximum of 0.48 kg kg^−1^), but DFE accumulation was not significantly correlated to any of the weather key figures. Instead, DFE accumulation (after blanching) was positively correlated with plant age, and biomass and positive correlations were observed between TSP and both illuminance and temperature, increasing the TSP in homogenates to 16 g kg^−1^ biomass. These observations were consistent with a previous study which reported higher protein expression in summer batches based on plant maturation up to a certain point (Sack et al., 2015). Others have reported the effects of light regimes (Goulet et al., 2019) and that short-term heat exposure (37°C) can boost transient protein expression (Norkunas et al., 2018).

Correlations (|r| > 0.70) were also observed between protease activity and internal relative humidity (negative correlation), as well as Temp_≥28_ and Ill_≥45,Av.tot._ (positive correlations) (**Table S2**), which is consistent with the reported heat-induced induction of plant protease activity (Bita and Gerats, 2013). These results suggested that, like the DFE yield, TSP was affected by the weather throughout cultivation but especially during sprouting, whereas protease activity was more sensitive to weather influences at the end of the cultivation and shortly before harvest. Interestingly, the amount of TSP varied without observable order and without correlation (r = −0.03) to DFE yield (**Figure S5**).

### Weather Data Cannot Explain Intra-batch Differences in Yield

The biochemical responses including DFE yield varied not only between batches but also across the three harvest times within one batch that were spaced by 1 week at the end of each cultivation period (**Figure 2B**, **Table S3**, **Figure S2**). We therefore analyzed the responses, such as DFE yield, for each batch individually, focusing on the final cultivation stage. We calculated the average values for all of the weather factors on the last day, over the last 2 days, over the last week, and over the last 2 weeks before harvest. For each batch, the DFE accumulation before and after blanching increased with subsequent harvests with only one exception (**Figure 2B**). We also correlated the weather factors with the relative yield at each harvest time. We defined this relative yield as the quotient of the yield at a given harvest time and the average yield of all harvests of the associated batch in order to normalize the yields across the different batches (second set of six batches, Equation 11). In three batches, the yield increased with each successive harvest point. For two batches, the second harvest was the most prolific, whereas most DFE was obtained from the first harvest in the remaining batch, and the second and third harvests yielded nearly the same quantities of product. These data suggest that the yield may increase up to an optimal harvest time and then decline (**Figure S6**), a similar pattern was also observed for recombinant monoclonal antibody 2G12 produced in transgenic tobacco plants (Sack et al., 2015). However, none of the weather factors correlated with this behavior around the time of harvest. We concluded that the differences in DFE yield between harvest times were not only caused by abiotic weather factors but probably also biotic changes such as the onset of anthesis or senescence. Accordingly, the variable DFE yield across different harvests probably reflected our limited ability to select a cultivation schedule that could compensate for seasonal effects on plant development in a greenhouse setting (**Figures 1E, F**) by adjusting the harvest time in the 37–63 dps range.

### A Linear Model Can Predict Average Batch Yields

We were interested in the generalizability of our findings and built a set of regression models linking the weather (e.g., Ill_≥45_) and cultivation (e.g., biomass) factors to DFE yield. Because scatterplots between weather and cultivation factors and yield showed a linear dependency, we selected a linear model that we limited to a maximum of two independent variables due to the small number of data points. A multilinear model based on Ill_≥45_ and plant age performed best (**Table 3**). The low values of the determination coefficients (<0.60) reflected the substantial scattering between different harvest times within batches, as discussed above.

**Table 3:** Multilinear regression models for DFE yield trained on all data or batch averages of the second set of six batches.

		Individual harvest data (n = 18)		Batch average data (n = 6)	
Independent variable 1	Independent variable 2	R^2^	adj. R^2^	R^2^	adj. R^2^
Ill_≥45_	Plant age	0.55	0.49	0.94	0.94
Ill_≥45_	Plant biomass	0.54	0.48	0.94	0.93
Inside temperature (total)	Plant age	0.54	0.48	0.90	0.88
Inside temperature (total)	Plant biomass	0.54	0.48	0.87	0.86
Ill_≥45_	Inside temperature (total)	0.53	0.46	0.93	0.92
Ill_≥45_	–	0.51	0.48	0.92	0.91
Inside temperature (total)	–	0.50	0.47	0.87	0.87

We therefore calculated the average DFE yields for each batch and found a strong correlation between the weather factors and the average DFE yield per batch—for example, r = −0.96 for Ill_≥45,S_. As expected, the corresponding *p*-values increased (reduced significance) compared to the model with individual harvests due to the lower number of data points (6 instead of 18) (**Table 1**). When the regression models were updated using the batch average DFE yield, they gave notably higher R^2^ and adjusted R^2^ values (**Table 3**). We used the best model trained with the data from the second set of six batches to calculate the DFE yield for the first set of six batches, which were not included in model training but achieved a poor prediction (R^2^ = 0.11). However, the correlation between the predicted and actual values of DFE yield was high (r = 0.87). The average yield of the first six batches was 1.69-fold higher than the average of the second set of six batches (**Figure S1D**). Using this value as a correction factor, we obtained a substantially higher coefficient of determination (R^2^ = 0.65) (**Figure 3C**). We assume that the offset between the two sets of batches was a sample treatment artifact because we froze the plants in the second set of batches allowing us to process them at the same time, whereas in the first set of batches, the plants were processed without freezing. Therefore, a simple linear model based on Ill_≥45_ and plant age can facilitate an *a priori* prediction of DFE yield from transgenic tobacco in a greenhouse setting.

## Conclusion

We observed a substantial effect of seasonal weather changes on the yield of the fusion protein DFE (based on the detection of an intact C-terminus) whereas its accumulation (based on fluorescence) was not affected by the greenhouse climate. Hence, care should be taken when assessing the suitability of growth conditions for the production of recombinant proteins in plants. In the future, controlled cultivation environments such as vertical farms (Wirz et al., 2012; Holtz et al., 2015) may help to reduce such seasonal effects and ensure consistent yields across batches. We found that high illuminance and/or high temperature, especially during the sprouting phase, reduced the yield of DFE, and this could also apply to other recombinant proteins. It was possible to predict the yield based on a simple model using illuminance and plant age. However, this model was not sufficient to calculate the effect of the harvest time on product yield and should thus be augmented to improve its predictive power—for example, by including additional factors that can describe the physiological development status of the plants.

## Data Availability Statement

The datasets generated for this study are available on request to the corresponding author.

## Author Contributions

MK conducted the experiments and collected the data. CR conducted the experiments and collected the data. JE analyzed the data and wrote the manuscript. JB devised the experiments, conducted the data analysis and wrote the manuscript.

## Funding

This work was funded in part by the Fraunhofer-Gesellschaft Internal Programs under Grant No. Attract 125-600164 and the state of North-Rhine-Westphalia under the Leistungszentrum grant no. 423 “Networked, adaptive production.” This work was supported by the Deutsche Forschungsgemeinschaft (DFG) in the framework of the Research Training Group “Tumor-targeted Drug Delivery” grant 331065168 and the European Research Council Advanced Grant “Future-Pharma,” proposal number 269110.

## Conflict of Interest

The authors declare that the research was conducted in the absence of any commercial or financial relationships that could be construed as a potential conflict of interest.

## Abbreviations

IMAC, immobilized metal-ion affinity chromatography; TSP, total soluble protein.

## Supplementary Material

Table S1 Correlation between climate factors during plant growth and DFE yield (as in **Table 1**) corrected for the influence of illuminance and temperature.

Table S2 Correlation coefficients between protease activity and weather key figures.

Table S3 Average DFE yields and standard deviations for the second set of six batches.

Figure S1 Climate and batch accumulation data for DFE. (A) Representative 10-day period during the cultivation of transgenic tobacco in a greenhouse setting (September 2016) illustrating the collinear course of illuminance and temperature. (B) Temperatures ≥28°C (zaxis) plotted against batch duration (x-axis) and DFE yield at the final harvest (y-axis). (C) Plot as in B but temperature replaced with illuminance ≥45 klx. (D) Box-plot of DFE yields in the first (2016) and second (2017) set of batches. Small open boxes indicate the set average. Boxes indicate the 25 and 75 quartiles, and whiskers mark the 5 and 95 percentiles.

Figure S2 Temperature (green) and illuminance (gray) curves recorded during the course of this study overlain with batch durations and dependent biochemical and product parameter results. The recovery is defined as the ratio of DFE in the elution fraction (after purification) and the DFE amount in the load (before purification). Horizontal lines indicate the average parameter value for that batch calculated based on all harvests of one batch, whereas colored point-scatter plots correspond to the individual harvest-specific values in the second batch set. Vertical colored dotted lines mark the transitions between the growth phases in each batch (left line = germination to sprouting, middle line = sprouting to growth, and right line = growth to maturation).

Figure S3 Correlations and cross-correlations between independent cultivation parameters observed for a second set of six batches (2.1–2.6). Three harvests spaced 1 week apart were conducted per batch resulting in a total of 18 data points (dots). Dots are colored according to the DFE yield after purification. Lines represent linear regression models for the parameters in the corresponding row and column and are colored according to their *p*-value: green = *p* < 0.01, orange = 0.01 < *p* < 0.05 and gray = *p* ≥ 0.05. Histograms in the diagonal of panels represent the distribution of the parameter defined by the corresponding row/column.

Figure S4 Correlation between selected independent cultivation parameters and dependent biochemical and product parameters observed for a second set of six batches (2.1–2.6). Three harvests spaced 1 week apart were conducted per batch resulting in a total of 18 data points (dots). Dots are colored according to the DFE yield after purification. Lines represent linear regression models for the parameters in the corresponding row and column and are colored according to their *p*-value: green = *p* < 0.01, orange = 0.01 < *p* < 0.05 and gray = *p* ≥ 0.05.

Figure S5 Correlation between dependent biochemical and product parameters observed for a second set of six batches (2.1–2.6). Three harvests spaced 1 week apart were conducted per batch resulting in a total of 18 data points (dots). Dots are colored according to the DFE yield after purification. Lines represent linear regression models for the parameters in the corresponding row and column and are colored according to their p-value: green = p < 0.01, orange = 0.01 < p < 0.05 and gray = p ≥ 0.05. Histograms in the diagonal of panels represent the distribution of the parameter defined by the corresponding row/column.

Figure S6 Relative yield in dependence of harvest time. (A) Relative yield (ry) of DFE calculated using Equation 11 for each harvest of the second set of plant batches. (B) Relative yield of DFE with the harvest time adjusted so that the maximum yield was at time zero. Interestingly, no “U”-shaped sequence of yields was observed. The plot may be used to identify optimal cultivation times in dependence of the season and weather conditions. For example longer cultivation may result in higher DFE yields for batch 2.2 (> 55 dps), 2.4 (> 52 dps) and 2.5 (> 63 dps), whereas an optimal harvest was identified for 2.1 (50 dps) and 2.5 (56 dps).
