# Supplementary material for: Seasonal Weather Changes Affect the Yield and Quality of Recombinant Proteins Produced in Transgenic Tobacco Plants in a Greenhouse Setting
Source: Front Plant Sci. 2019 Oct 8;10:1245. doi: 10.3389/fpls.2019.01245 (PMC6791924; doi:10.3389/fpls.2019.01245)
Supplement: Table S1 — Correlation between climate factors during plant growth and DFE yield (as in Table 1 ) corrected for the influence of illuminance and temperature. [file DataSheet_1.pdf]

Title:

Seasonal weather changes affect the yield and quality of recombinant proteins produced in transgenic tobacco plants in a greenhouse setting

M. Knödler[1,2], C. Rühl[1], J. Emonts[1], J. F. Buyel[1,2,*]

[1]Fraunhofer Institute for Molecular Biology and Applied Ecology IME, Forckenbeckstraße 6, 52074 Aachen, Germany

[2]Institute for Molecular Biotechnology, Worringerweg 1, RWTH Aachen University, 52074 Aachen, Germany

Supplementary data

Table S1. Correlation between climate factors during plant growth and DFE yield (as in Table 1) corrected for the influence of illuminance and temperature.

				24 h		Light period[a]		Dark period[b]	
Data type	Sensor location	Weather factor	Unit	r	p-value	r	p-value	r	p-value
All data	Inside	Temperature	[°C]	-0.170	0.514	-0.184	0.480	-0.074	0.779
		Temperature change	[°C]	-0.226	0.384	-0.230	0.375	-0.106	0.687
		Illuminance	[klx]	-0.120	0.647	-0.120	0.647	n.a.	n.a.
		Relative humidity	[-]	0.034	0.897	0.070	0.789	-0.056	0.832
	Outside	Temperature	[°C]	0.139	0.594	0.140	0.593	0.129	0.621
		Relative humidity	[-]	0.087	0.740	0.108	0.681	0.052	0.844
		Rain	[m]	0.238	0.357	0.256	0.321	0.191	0.463
		Wind	[m s^{-1}]	-0.191	0.463	-0.193	0.457	-0.177	0.496
Averages	Inside	Temperature	[°C]	-0.404	0.500	-0.433	0.466	-0.173	0.780
		Temperature change	[°C]	-0.557	0.330	-0.576	0.309	-0.037	0.953
		Illuminance	[klx]	-0.298	0.626	-0.298	0.626	n.a.	n.a.
		Relative humidity	[-]	0.135	0.829	0.226	0.714	-0.099	0.875
	Outside	Temperature	[°C]	0.359	0.553	0.372	0.538	0.297	0.628
		Relative humidity	[-]	0.452	0.445	0.518	0.371	0.336	0.580
		Rain	[m]	0.713	0.177	0.760	0.136	0.590	0.295
		Wind	[m s^{-1}]	-0.433	0.466	-0.452	0.445	-0.373	0.536

Table S2. Correlation coefficients between protease activity and weather key figures.

	Inside relative humidity (light period)		$Temp_{\geq 28}$ (maturation)		$Ill_{\geq 45}$ (growth)		$Ill_{\geq 45}$ (maturation)	
	r	p-value	r	p-value	r	p-value	r	p-value
Protease activity (before blanching)	-0.782	0.000	0.733	0.001	0.696	0.001	0.645	0.004
Protease activity (after blanched)	-0.790	0.000	0.612	0.007	0.749	0.000	0.656	0.003

Table S3. Average DFE yields and standard deviations for the second set of six batches.

	Batch 2.1	Batch 2.2	Batch 2.3	Batch 2.4	Batch 2.5	Batch 2.6
Average yield	9.31	1.52	8.42	8.41	12.26	15.99
Standard deviation	2.65	1.09	1.54	5.07	7.61	6.47

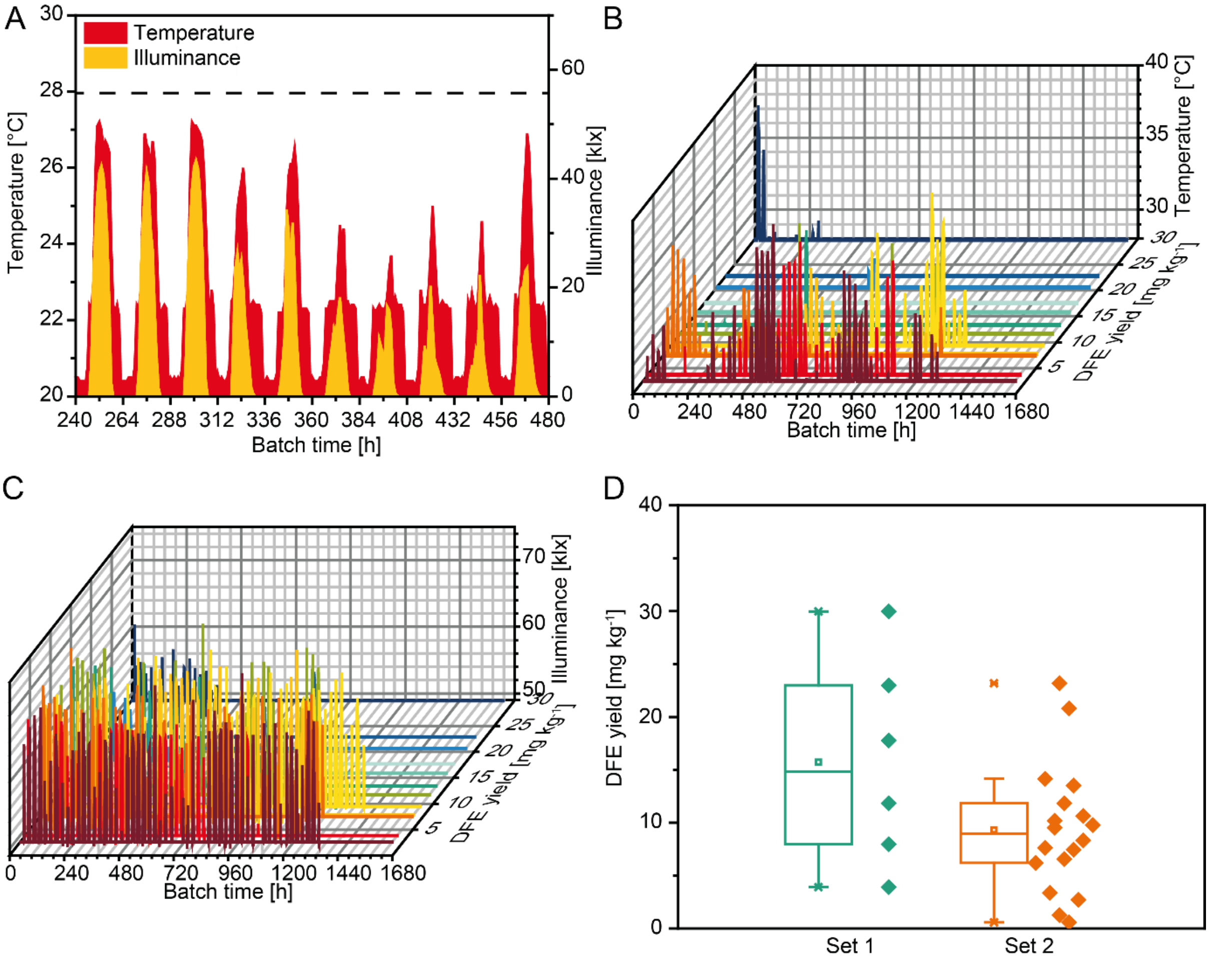

Figure S1. Climate and batch accumulation data for DFE. **(A)** Representative 10-day period during the cultivation of transgenic tobacco in a greenhouse setting (September 2016) illustrating the collinear course of illuminance and temperature. **(B)** Temperatures ≥28°C (z-axis) plotted against batch duration (x-axis) and DFE yield at the final harvest (y-axis). **(C)** Plot as in B but temperature replaced with illuminance ≥45 klx. **(D)** Box-plot of DFE yields in the first (2016) and second (2017) set of batches. Small open boxes indicate the set average. Boxes indicate the 25 and 75 quartiles, and whiskers mark the 5 and 95 percentiles.

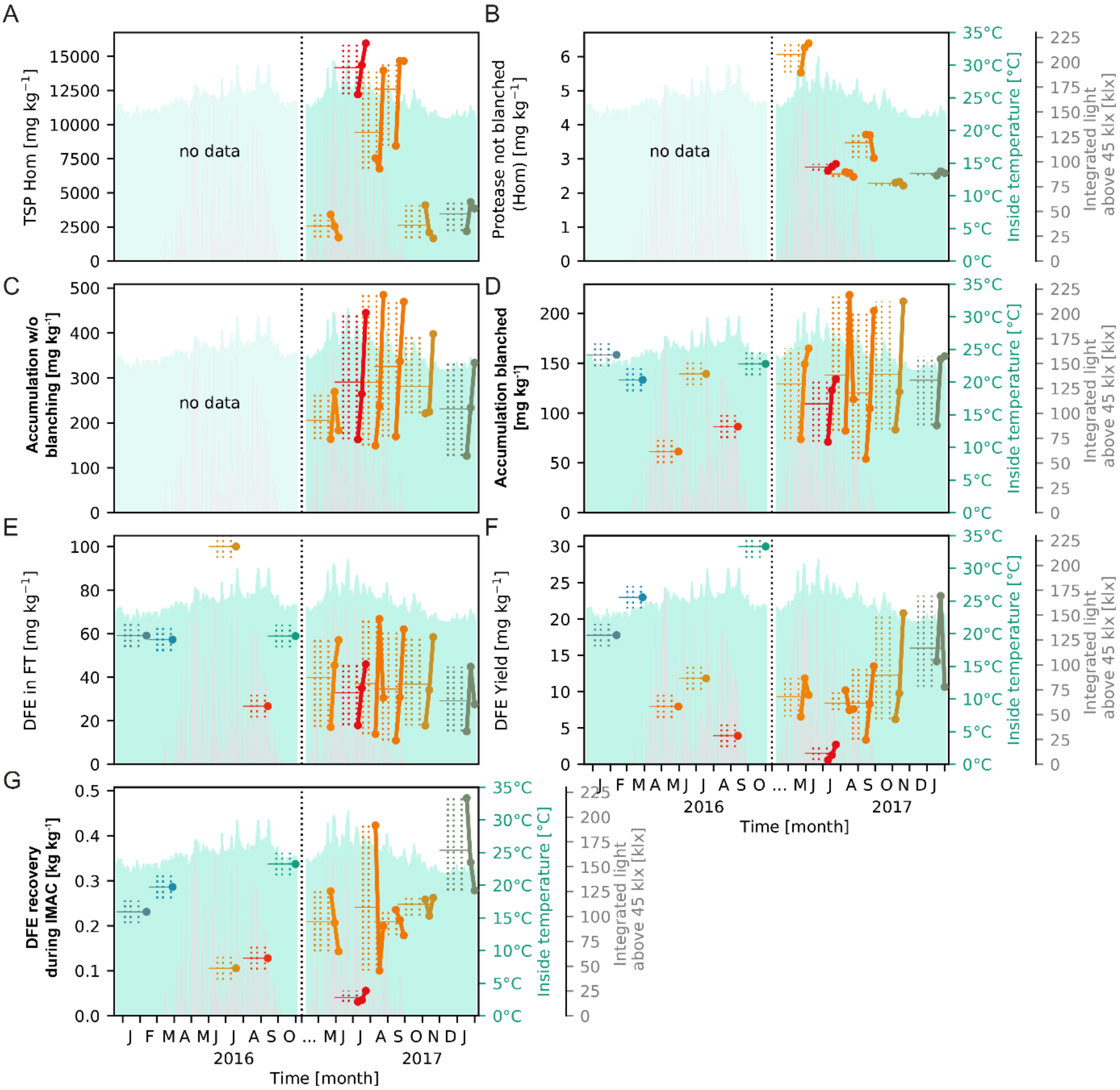

Figure S2: Temperature (green) and illuminance (gray) curves recorded during the course of this study overlain with batch durations and dependent biochemical and product parameter results. **(A)** Total soluble protein (TSP) measured in leaf extracts. **(B)** Protease activity in extracts of plant leaves that were blanched (submersed in hot buffer) before homogenization. **(C)** DFE concentration in untreated leaf biomass as measured by fluorescence. **(D)** DFE concentration in leaves that were blanched before extraction. **(E)** DFE concentration in the flow-through fraction of IMAC chromatography, i.e. DFE that did not bind to the resin. **(F)** DFE yield per kilogram of biomass after the final purification step. **(G)** Step recovery of IMAC purification as the ratio of loaded to recovered DFE. The recovery is defined as the ratio of DFE in the elution fraction (after purification) and the DFE amount in the load (before purification). Horizontal lines indicate the average parameter value for that batch calculated based on all harvests of one batch, whereas colored point-scatter plots correspond to the individual harvest-specific values in the second batch set. Vertical colored dotted lines mark the transitions between the growth phases in each batch (left line = germination to sprouting, middle line = sprouting to growth, and right line = growth to maturation).

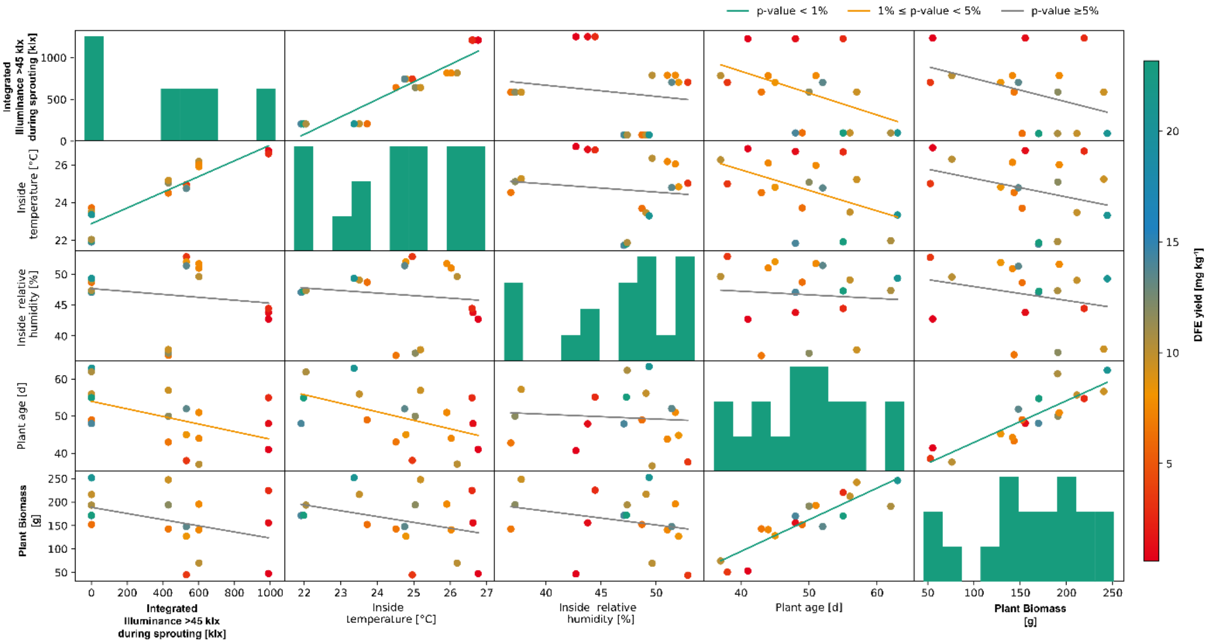

Figure S3. Correlations and cross-correlations between independent cultivation parameters observed for a second set of six batches (2.1–2.6). Three harvests spaced 1 week apart were conducted per batch resulting in a total of 18 data points (dots). Dots are colored according to the DFE yield after purification. Lines represent linear regression models for the parameters in the corresponding row and column and are colored according to their *p*-value: green = $p<0.01$, orange = $0.01<p<0.05$ and gray = $p\geq0.05$. Histograms in the diagonal of panels represent the distribution of the parameter defined by the corresponding row/column.

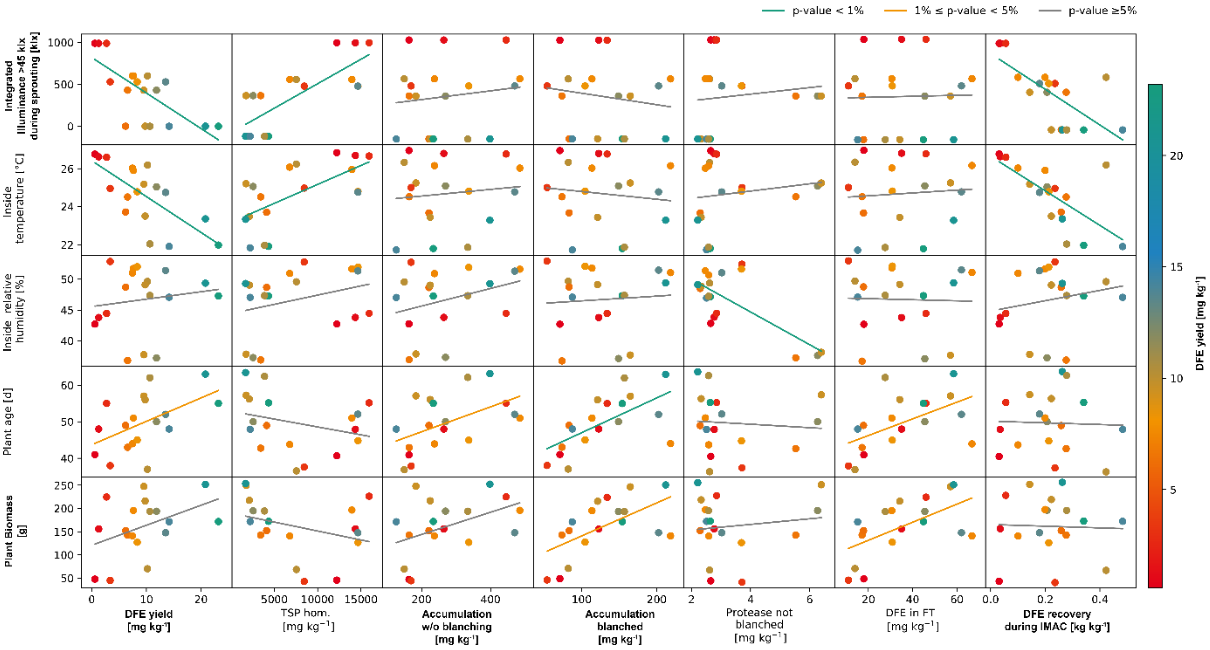

Figure S4. Correlation between selected independent cultivation parameters and dependent biochemical and product parameters observed for a second set of six batches (2.1–2.6). Three harvests spaced 1 week apart were conducted per batch resulting in a total of 18 data points (dots). Dots are colored according to the DFE yield after purification. Lines represent linear regression models for the parameters in the corresponding row and column and are colored according to their *p*-value: green = $p<0.01$, orange = $0.01<p<0.05$ and gray = $p\geq0.05$.

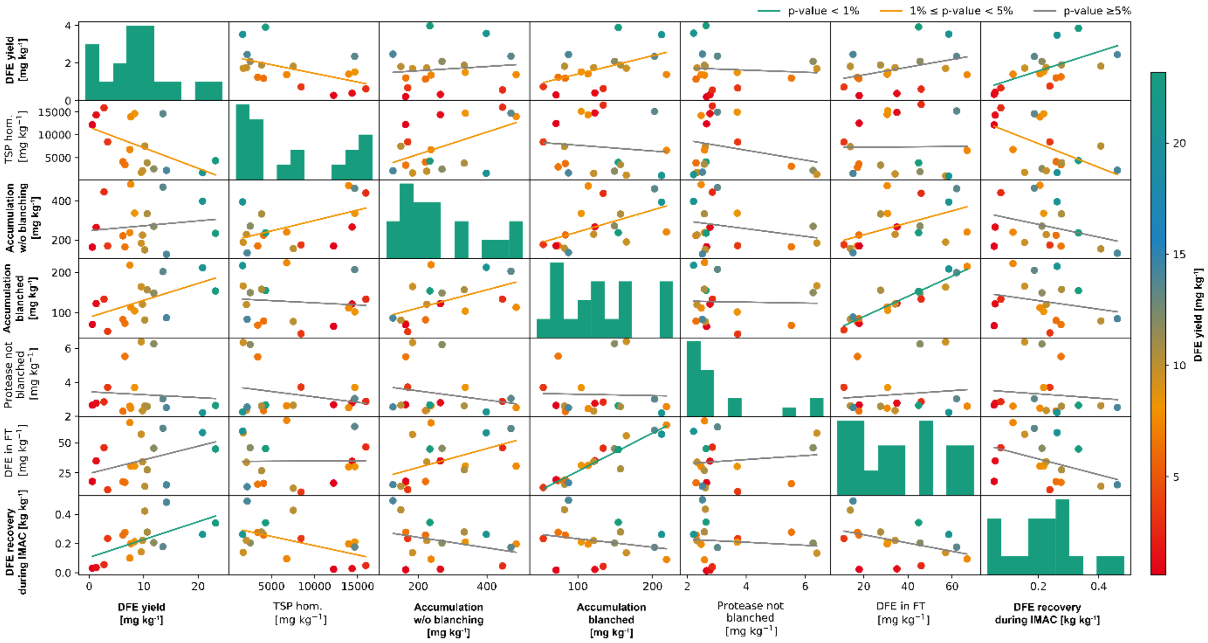

Figure S5. Correlation between dependent biochemical and product parameters observed for a second set of six batches (2.1–2.6). Three harvests spaced 1 week apart were conducted per batch resulting in a total of 18 data points (dots). Dots are colored according to the DFE yield after purification. Lines represent linear regression models for the parameters in the corresponding row and column and are colored according to their *p*-value: green = $p<0.01$, orange = $0.01<p<0.05$ and gray = $p\geq0.05$. Histograms in the diagonal of panels represent the distribution of the parameter defined by the corresponding row/column.

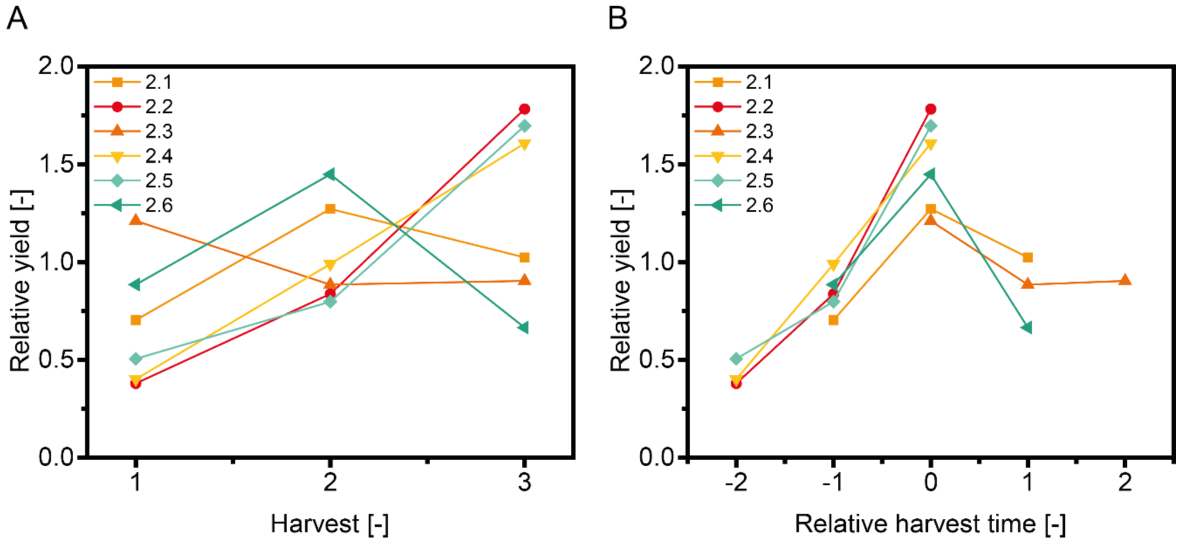

Figure S6. Relative yield in dependence of harvest time. **(A)** Relative yield (ry) of DFE calculated using **Equation 11** for each harvest of the second set of plant batches. **(B)** Relative yield of DFE with the harvest time adjusted so that the maximum yield was at time zero. Interestingly, no "U"-shaped sequence of yields was observed. The plot may be used to identify optimal cultivation times in dependence of the season and weather conditions. For example longer cultivation may result in higher DFE yields for batch 2.2 (>55 dps), 2.4 (>52 dps) and 2.5 (>63 dps), whereas an optimal harvest was identified for 2.1 (50 dps) and 2.5 (56 dps).
